# Shared and Specific Intrinsic Functional Connectivity Patterns in Unmedicated Bipolar Disorder and Major Depressive Disorder

**DOI:** 10.1038/s41598-017-03777-8

**Published:** 2017-06-15

**Authors:** Ying Wang, Junjing Wang, Yanbin Jia, Shuming Zhong, Meiqi Niu, Yao Sun, Zhangzhang Qi, Ling Zhao, Li Huang, Ruiwang Huang

**Affiliations:** 1 Medical Imaging Center, First Affiliated Hospital of Jinan University, Guangzhou, 510630 China; 2 Clinical Experimental Center, First Affiliated Hospital of Jinan University, Guangzhou, 510630 China; 3 Center for the Study of Applied Psychology & MRI Center, Key Laboratory of Mental Health and Cognitive Science of Guangdong Province, School of Psychology, Institute of Brain Research and Rehabilitation, South China Normal University, Guangzhou, 510631 China; 4 Department of Psychiatry, First Affiliated Hospital of Jinan University, Guangzhou, 510630 China

## Abstract

Identifying brain differences and similarities between bipolar disorder (BD) and major depressive disorder (MDD) is necessary for increasing our understanding of the pathophysiology and for developing more effective treatments. However, the features of whole-brain intrinsic functional connectivity underlying BD and MDD have not been directly compared. We collected resting-state fMRI data from 48 BD patients, 48 MDD patients, and 51 healthy subjects. We constructed voxel-wise whole-brain functional networks and computed regional functional connectivity strength (FCS) using graph-theory and further divided the regional FCS into long-range FCS (lFCS) and short-range FCS (sFCS). Relative to the controls, both the BD and MDD patients showed decreased sFCS in the bilateral precuneus. In addition, the BD patients showed increased and the MDD patients showed decreased lFCS and sFCS in the bilateral cerebellum. The BD patients also showed increased lFCS in the right middle temporal gyrus and increased sFCS in the bilateral thalamus compared to either the MDD patients or the controls. These findings suggest that BD and MDD may have some shared as well as a greater number of specific impairments in their functional connectivity patterns, providing new evidence for the pathophysiology of BD and MDD at the large-scale whole brain connectivity level.

## Introduction

Bipolar disorder (BD) and major depressive disorder (MDD, or unipolar depression) are highly prevalent and debilitating conditions, which are associated with high suicide rates and impose a heavy social and economic burden^1^. Because BD often presents clinically as a depressive episode, many patients with this disorder are misdiagnosed^2^, leading to inappropriate treatment and poor clinical outcomes^3^. Consequently, the proper and reliable diagnosis of BD and MDD poses a major challenge for clinicians, particularly when recurring episodes of depression are the primary common affective symptoms in both disorders. Identifying biomarkers based on neuroimaging techniques will not only facilitate more accurate differential diagnosis but will also advance our understanding of the pathological mechanisms underlying mood disorders.

Direct comparisons between BD and MDD of neuroimaging measures are sparse and the results are inconsistent. A meta-analysis of structural neuroimaging studies found that MDD patients had increased volume in the corpus callosum but decreased volume in the hippocampus and basal ganglia compared with BD patients^4^. A recent major step forward in the field has been the realization that distributed brain connectivity rather than individual regions could underlie the pathophysiology of psychotic disorders^5^. Diffusion tensor imaging (DTI) studies reported more widespread white matter connectivity abnormalities in BD relative to MDD^2, 6, 7^. Intrinsic functional connectivity, a measure derived from resting-state fMRI (R-fMRI), has emerged as an effective tool for exploring large-scale human brain organization. Independent research on BD and MDD have increasingly emphasized a role for dysconnectivity in large-scale brain networks in these two disorders, particularly in the default mode network (DMN) and prefrontal-limbic network^8–10^. In addition, previous methods calculated functional connectivity between brain regions by using seed-based correlation analysis and independent component analysis but have not measured the total number of functional connectivity per voxel.

Given the complex etiology and symptomatology of BD and MDD as well as the wide variety of brain structural and functional abnormalities reported^11, 12^, we proposed that it would be of great interest to examine the topology of the voxel-wise functional connectivity network throughout the entire brain. Whole-brain functional connectivity strength (FCS) mapping, a data-driven graph theory approach, measures the number of functional connections between a given voxel and other voxels^13–15^. Several studies have demonstrated that the FCS metric is closely associated with physiological measures such as regional cerebral blood flow^14^, aerobic glycolysis^16^, and oxidative glucose metabolism^15^. In fact, FCS alterations and correlations with pathophysiologic mechanisms have also been demonstrated in MDD^17–19^, Alzheimer disease^20^, schizophrenia^21^, and obsessive compulsive disorder^22^. Additionally, the effect of spatial distance on functional connectivity was further studied by dividing the global FCS into long- and short-range FCS according to the anatomical distance of a given voxel^23, 24^. The efficient, well-organized functioning of a human brain depends on both long- and short-range connections^25^. Long-range functional connectivity runs at higher metabolic and time costs^14, 26^, whereas short-range functional connectivity runs at lower metabolic and time costs. For instance, a developmental study of the healthy brain revealed that long-range and short-range functional connectivity increases and decreases with age, respectively^27^. Until now, no study has directly compared the difference in distance-related functional connectivity between MDD and BD.

In this study, we constructed voxel-wise whole-brain functional networks in BD and MDD patients as well as a group of healthy controls using R-fMRI data and performed long- and short-range FCS analyses. The goal was to study the whole-brain voxel-wise intrinsic functional connectivity patterns in unmedicated BD and MDD patients during a depressive episode. We hypothesized that BD and MDD patients would show shared and specific patterns in the disrupted topological organization of the functional connectome, especially in the DMN and the limbic network.

## Methods and Materials

### Subjects

A total of 53 currently depressed adults diagnosed with BD II and 56 currently depressed adults diagnosed with MDD were recruited from the Psychiatry Department of the First Affiliated Hospital of Jinan University, Guangzhou, China. The diagnoses of BD and MDD were made according to the Structured Clinical Interview for DSM-IV. The clinical state of each patient was assessed using the 24-item Hamilton Depression Rating Scale (HAMD) and the Young Mania Rating Scale (YMRS) during the seven-day period prior to the R-fMRI scan. The inclusive criteria were a HAMD-24 total score > 21 for the MDD patients and a YMRS total score < 7 and a HAMD-24 total score > 21 for the BD patients^28^. The exclusion criteria were patients with other Axis-I psychiatric disorders (except for UD, BD, and anxiety disorders), a history of organic brain disorder, neurological disorders, mental retardation, cardiovascular diseases, alcohol or substance abuse, pregnancy, or any physical illness. At the time of testing, all patients were either medication-naive or had been unmedicated for at least five months. None of the patients had received psychotherapy or electroconvulsive therapy prior to participating in the study.

In addition, we recruited 54 sex-, age-, and education-matched healthy subjects as controls via local advertisements. They were carefully screened through a diagnostic interview, the Structured Clinical Interview for DSM-IV (nonpatient edition), to rule out the presence of current or past psychiatric illness. Further exclusion criteria for the healthy controls were any history of psychiatric illness in first-degree relatives and any current or past significant medical illness or mental disorders.

All subjects were right-handed according to their self-report. The study was approved by the Ethics Committee of the First Affiliated Hospital of Jinan University, China, and the methods and procedures were carried out in accordance with the approved guidelines. All subjects signed a written informed consent form after a full written and verbal explanation of the study. Two senior clinical psychiatrists confirmed that all subjects had the ability to consent to participate in the examination.

### Data acquisition

All MRI datasets were obtained on a 3 T GE MR750 scanner with an eight-channel phased-array head coil in the Medical Center of the First Affiliated Hospital of Jinan University, Guangzhou. The R-fMRI datasets were acquired using a single-shot gradient-echo EPI sequence with the following parameters, repetition time (TR) = 2,000 ms, echo time (TE) = 25 ms, flip angle (FA) = 90°, field of view (FOV) = 240 mm × 240 mm, data matrix = 64 × 64, thickness/gap = 3.0/1.0 mm, 35 axial slices covering the whole-brain, and 210 volumes acquired in 7 minutes. During the R-fMRI scan, each subject was asked to keep their eyes closed but not to fall asleep, and to relax the mind but not to think about anything in particular. In addition, routine axial T1-weighted fluid attenuation inversion recovery (FLAIR) and fast spin-echo T2-weighted MR sequences were also applied to obtain brain images to confirm the absence of brain structural and signal abnormality.

### Data preprocessing

The R-fMRI data were preprocessed using SPM8 (http://www.fil.ion.ucl.ac.uk/spm/) and DPARSF (http://restfmri.net/forum/DPARSF). For each subject, the first ten volumes of the R-fMRI dataset were discarded to allow for MR signal equilibrium, leaving 200 volumes for further analyses. The remaining functional images were first corrected for the acquisition time delay between slices within the same TR and then were realigned to the first volume for correcting for inter-TR head motion. This realignment calculation provided a record of the head motion within the R-fMRI scan. All of the subjects in this study satisfied our criteria for head motion, displacement < 1.5 mm in any plane and rotation < 1.5° in any direction. In addition, there was no difference in the mean frame-wise displacement of head motion between the three groups (*p* = 0.46). The R-fMRI data were spatially normalized to Montreal Neurological Institute (MNI) space, and were resampled to a voxel size of 3 × 3 × 3 mm^3^. The waveform for each voxel was detrended and passed through a band-pass filter of 0.01–0.08 Hz to reduce the effects of low-frequency drift and high-frequency physiological noise. Finally, we regressed out nuisance covariates from each voxel’s time course, including the signals of the brain white matter and cerebrospinal fluid (CSF), as well as the Friston-24 parameters of head motion.

### Voxel-wise weighted FCS

For each subject, we constructed a voxel-wise brain functional network with a node representing a voxel and the inter-voxel temporal correlation of the BOLD signals representing the edge weights. For a given node, we quantified its importance in a network by using the weighted FCS, which is defined as the summation of the connectivity strengths of all the connections between that node and all the other nodes^17, 19, 29^. The FCS for a given voxel *i* is defined by ref. 18

1 $$s(i)={\sum }_{j=1}^{N}{r}_{ij}-1,$$

where *r*_*ij*_ is the Pearson’s correlation coefficient between voxels *i* and *j* in their BOLD signal time series and *N* is the number of grey matter (GM) voxels according to the GM probability map in SPM8 (GM probability > 20%). To examine the effects of anatomical distance on FCS analyses, we divided the FCS into short-range FCS (sFCS) and long-range FCS (lFCS)^23, 24^. The sFCS of a voxel is defined as the sum of the correlations between the given voxel and other voxels with an anatomical distance less than 75 mm, while the lFCS of a voxel is defined as the sum of the connections with an anatomical distance greater than 75 mm^23, 24^. Here, the anatomical distance between two voxels refers to the Euclidean distance measured using their MNI coordinates. In addition, we employed a thresholding procedure based on the statistical significance level of the correlation analyses to avoid the contamination of spurious weak correlations. Specifically, we first calculated the corresponding *p*-values of all the elements in each correlation matrix. Each *p*-value is the probability of getting a correlation as large as the observed value by random chance when the true correlation is zero. Then the inter-nodal temporal correlations surviving at a threshold of *p* < 0.05 (Bonferroni corrected) were retained or were reset to 0. In the calculations, we also set the negative inter-nodal temporal correlations as 0 given their ambiguous interpretation and the detrimental effects of test-retest reliability^30–33^. In the end, two types of FCS maps, lFCS and sFCS maps, were obtained for each subject.

## Statistical analyses

### Group differences

For each subject, we first normalized the two types of FCS maps with a Fisher’s *r*-to-*z* transformation based on the mean and SD across all voxels in each FCS map and then smoothed them with a Gaussian kernel of full-width at half maximum = 4 mm. Thus, we obtained the *sz*-lFCS and *sz*-sFCS maps for each subject. For convenience, in the following analyses, we used the lFCS and sFCS to refer to the *sz*-lFCS and *sz*-sFCS, respectively (also in Figures and Tables). Statistical tests were performed on each of the FCS maps across the three groups using a voxel-wise one-way analysis of covariance (ANCOVA) with age, gender, and the mean frame-wise displacement of head motion as the covariates. The result was corrected for multiple comparisons using the AlphaSim program based on the Monte Carlo simulation algorithm^34^. The clusters with significant group differences in each type of FCS map were determined at a threshold of *p* < 0.001 (Alphasim corrected), which was implemented in DPARBI by combining a height threshold and an extent threshold determined by Monte Carlo simulations^35^. For each of the detected clusters, we extracted the mean FCS of a spherical ROI with the centroid at its corresponding peak voxel (radius = 4 mm) and then conducted the post hoc analysis. When we performed the post hoc analysis comparing the BD and MDD groups, we also controlled for the age of onset as one of the covariates.

### Brain-behavioral relationship

For the clusters showing significant group effects (one-way ANCOVA), we performed partial correlation analyses to describe the relationships between the lFCS or sFCS and the clinical variables in the BD and MDD groups, separately (*p* < 0.05, Bonferroni corrected). The clinical variables included the HAMD score, number of episodes, onset age of illness, and total duration of illness. In the calculations, we controlled for the age, gender, and the mean frame-wise displacement of head motion as the covariates.

## Classification

We also plotted the receiver operating characteristic (ROC) curves to determine which of these clusters with significant group effects (ANCOVA) in lFCS or sFCS could be clearly used to distinguish the BD patients from the controls, the MDD patients from the controls, as well as the BD from the MDD patients. The ROC curve, a fundamental plot in signal detection theory that is widely used in medical science, is a scatter plot showing the relationship between false alarm rates and hit rates. It describes the relationship between the underlying distribution of the places where signals are absent and the places where signals are present. This analysis applied a public MATLAB code (http://www.mathworks.com/matlabcentral/fileexchange/.

199500roc-curve; by Giuseppe Cardillo).

## Results

### Demographic information

The demographic and clinical characteristics of the three groups are given in Table 1. Five patients with BD, eight patients with MDD, and three control participants were excluded from further analyses because of excessive head motion or partial cerebellum coverage of their MRI scans. Finally, the participants included 48 patients with BD, 48 patients with MDD and 51 healthy control subjects. There was no significant differences between the three groups in gender, age, or education. Additionally, there was no significant differences in the HAMD scores (*p* = 0.21), YRMS scores (*p* = 0.47), number of episodes, and illness duration between the two depressed groups. However, the age of onset of the BD patients was significantly earlier than that of the MDD patients (*p* < 0.05).

**Table 1:** Demographics and clinical characteristics of the BD patients, MDD patients, and healthy controls in this study. Abbreviations: BD (MDD), bipolar (major depressive) disorder; HAMD, Hamilton Depression Rating Scale; YRMS, Yong Mania Rating Scale.

Parameters	BD (*n* = 48)	MDD (*n* = 48)	Controls (*n* = 51)	*p*-value
Age (years old)	27.33 ± 8.41	29.67 ± 8.44	29.94 ± 11.18	0.33^a^
Gender (female/male)	23/25	28/20	31/20	0.40^b^
Education (years)	14.31 ± 2.54	13.40 ± 2.89	14.27 ± 2.83	0.18^a^
Number of Episodes	2.54 ± 1.65	2.02 ± 1.87	N/A	0.09^c^
Age of Onset (years)	23.00 ± 9.33	26.60 ± 9.21	N/A	0.03^c^
Duration of Illness (months)	43.13 ± 54.53	32.66 ± 44.70	N/A	0.15^c^
HAMD	26.19 ± 5.70	25.27 ± 5.17	2.88 ± 1.80	0.00^a^
YRMS	2.96 ± 4.07	3.04 ± 3.70	0.47 ± 0.90	0.00^a^

^a^The *p*-value was obtained from permutation ANOVA analysis.

^b^The *p*-value was estimated obtained from Pearson’s *χ* ^2^-test.

^c^The *p*-value was calculated from permutation two-sample *t* test.

N/A, not applicable.

### FCS maps

The FCS maps are presented in Figures S1 and S2 (Supplementary materials). A visual examination indicated that the spatial distribution of brain clusters with high FCS were remarkably similar in locations across the three subject groups in spite of some differences in their height and size. For the lFCS, the clusters with high connections were predominantly in the bilateral inferior temporal gyri (ITG), middle temporal gyri (MTG), medial frontal gyri (MFG), inferior parietal gyri (IPG), and posterior cingulate cortex (PCC). For the sFCS, the clusters with high connections were located in the bilateral middle occipital gyri (MCG), inferior parietal gyri (IPG), PCC, and cerebellum.

### Group effect in lFCS

**Figure 1:**
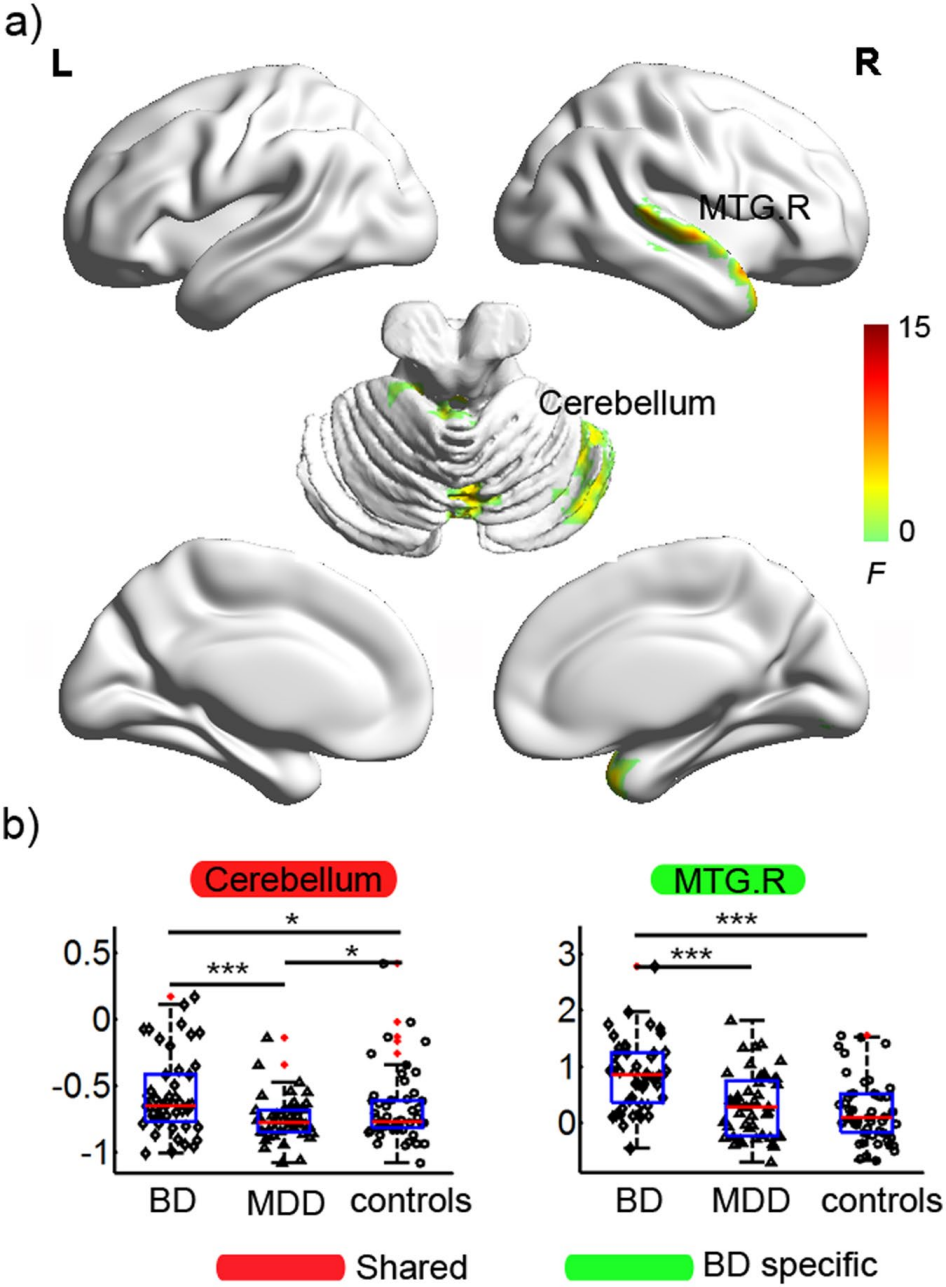
Statistical results for the lFCS in the BD, MDD, and healthy controls. (**a**) Clusters showing significant group effects (*p* < 0.001 Alphasim corrected, ANCOVA). The brain surfaces were visualized using BrainNet Viewer (http://www.nitrc.org/projects/bnv/). (**b**) Post-hoc analyses of the lFCS for these significant clusters. The ‘BD’ type indicates that the lFCS in the cluster was specifically altered in the BD patients but not in the MDD patients. The ‘shared’ type indicates that the cluster with altered lFCS was detected in both the BD and MDD patients. ◊, ∆, and ○ in the scatter plot indicate the mean lFCS values of a given cluster for a subject in the BD, MDD, and control groups, respectively. Red dots indicate outliers. The box plot shows the median (red line), interquartile range (blue lines), and sample minimum and maximum values (dark lines). The horizontal lines on top indicate pairwise comparisons that survived statistical thresholds: ***p < 0.001; **p < 0.01; *p < 0.05. Abbreviations: lFCS, long-range functional connectivity strength; BD (MDD), bipolar (major depressive) disorder; MTG, middle temporal gyrus; B (R), bilateral (right).

Figure 1a displays the brain clusters, including the right MTG and bilateral cerebellum (including the anterior, posterior, and vermis areas), showing a significant group effect in lFCS. Post hoc analyses (Fig. 1b) showed that the BD group showed significantly increased lFCS, but the MDD group showed significantly decreased lFCS in the cerebellum compared to the control group. Moreover, the BD group showed significantly increased lFCS in the right MTG compared to either the MDD or the control group.

### Group effect in sFCS

**Figure 2:**
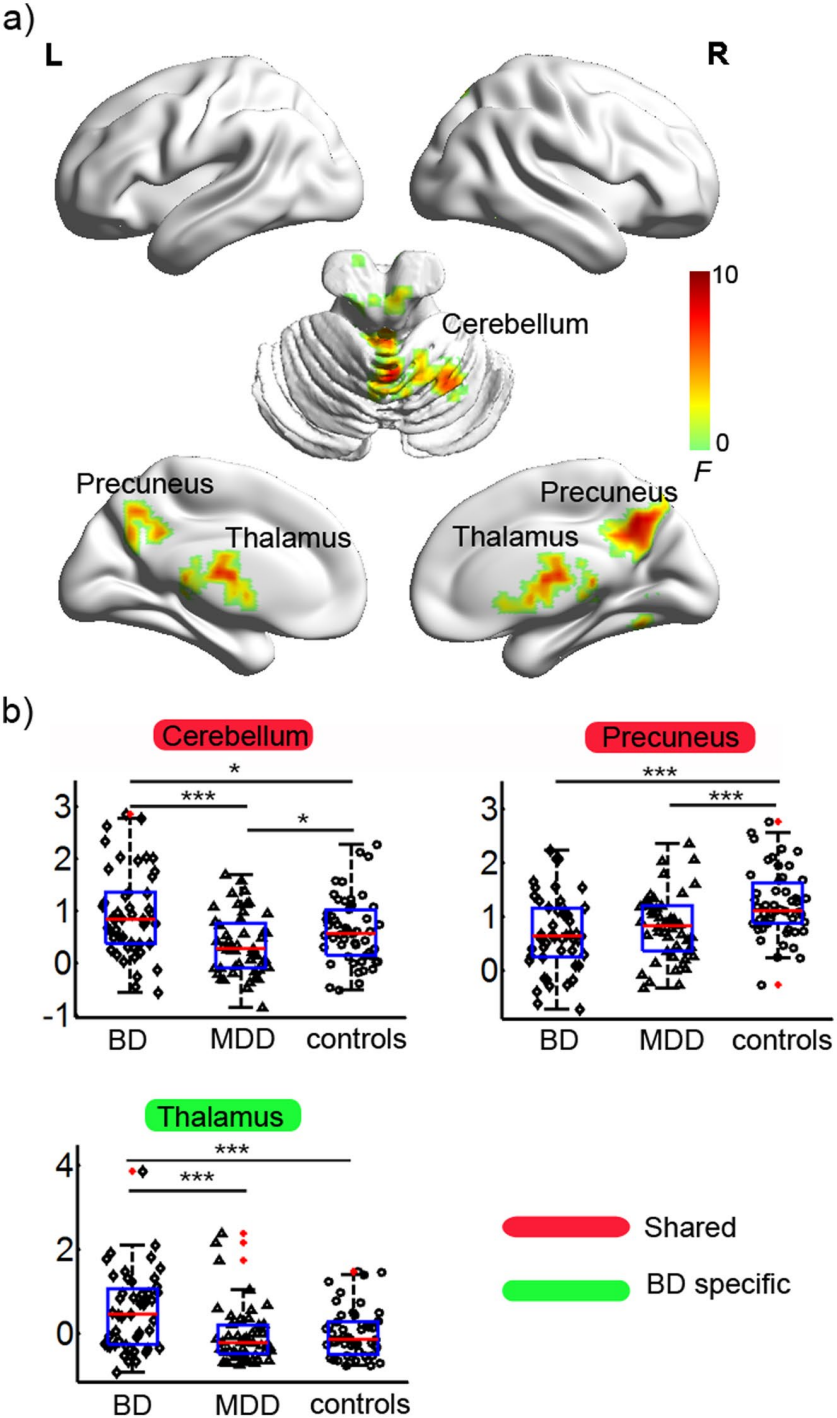
Statistical results for the sFCS in the BD, MDD, and healthy controls. (**a**) Clusters showing significant group effects (*p* < 0.001 Alphasim corrected, ANCOVA). The brain surfaces were visualized using BrainNet Viewer (http://www.nitrc.org/projects/bnv/). (**b**) Post-hoc analyses of the sFCS for the significant clusters. The ‘BD’ type indicates that the sFCS in the cluster was specifically altered in the BD patients but not in the MDD patients. The ‘shared’ type indicates that the cluster with altered sFCS was detected in both the BD and the MDD patients. ◊, ∆, and ○ in the scatter plot indicate the mean lFCS values of a given cluster for a subject in the BD, MDD, and control groups, respectively. Red dots indicate outliers. The box plot shows the median (red line), interquartile range (blue lines), and sample minimum and maximum values (dark lines). The horizontal lines on top indicate pairwise comparisons that survived statistical thresholds: ***p < 0.001; **p < 0.01; *p < 0.05. Abbreviations: sFCS, short-range functional connectivity strength; BD (MDD), bipolar (major depressive) disorder; B (L, R), bilateral (left, right).

Figure 2a displays the brain clusters, including the bilateral precuneus, bilateral thalamus, and bilateral cerebellum (including the anterior and vermis areas) showing a significant group effect in sFCS. Post hoc analyses (Fig. 2b) showed that both the BD and MDD groups had significantly decreased sFCS in the bilateral precuneus compared to the control group. The BD group showed a significantly increased sFCS, but the MDD group showed a significantly decreased sFCS in the cerebellum compared to the control group. Moreover, the BD group showed a significantly increased sFCS in the bilateral thalamus compared to either the MDD or the control group.

### Brain-behavioral correlations

**Figure 3:**
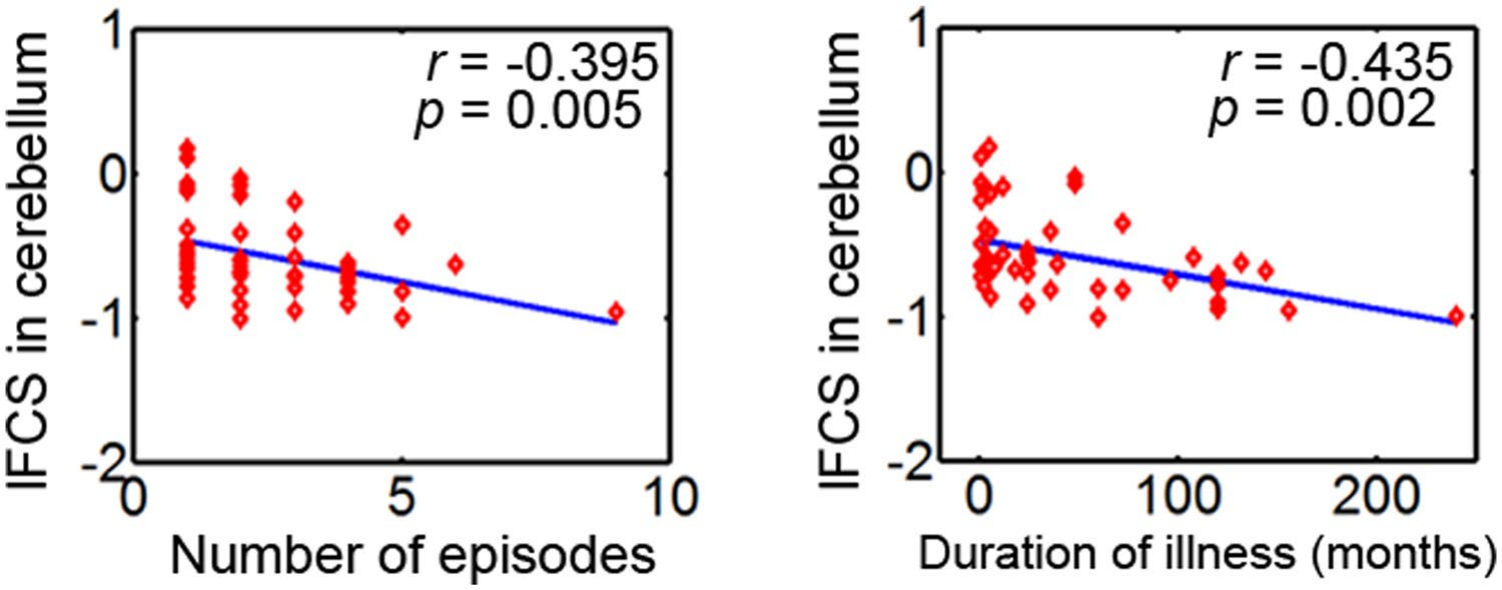
The relationship between the lFCS and clinical variables in the BD group (*p* < 0.05, Bonferroni corrected). The lFCS of the cerebellum was significantly negatively correlated with the number of episodes (*r* = −0.395, *p* = 0.005) and the duration of illness (*r* = −0.435, *p* = 0.002).

For all the clusters listed in Table 2, we performed partial correlation analyses to describe the relationships between lFCS or sFCS and the clinical variables in the BD and MDD groups (*p* < 0.05, Bonferroni corrected). In the BD group, the lFCS of the cerebellum were significantly negatively correlated with the number of episodes (*r* = −0.395, *p* = 0.005) and duration of illness (*r* = −0.435, *p* = 0.002) (Fig. 3). However, no significant correlation was found between the FCS and any of the clinical variables in the MDD group.

**Table 2:** Brain clusters showing significant group effect in the FCS (*p* < 0.001 Alphasim corrected, ANCOVA). The ‘BD’ type indicates that the FCS in the cluster was altered specifically in the BD patients but not in the MDD patients. The ‘shared’ type indicates that the cluster with altered FCS was detected in both the BD and the MDD patients. Abbreviations: lFCS (sFCS), long-range (short-range) functional connectivity strength; BD (MDD), bipolar (major depressive) disorder; MTG, middle temporal gyrus; B (R), bilateral (right).

FCS	Cluster	Type	Location	Cluster size (voxels)	MNI coordinates (x, y, z)	*F-*value
lFCS						
	R MTG	BD	BA21	317	66, −27, −3	15.02
	Cerebellum	Shared	—	541	9, 60, −33	7.84
sFCS						
	B Thalamus	BD	—	306	−6, −15, 12	9.14
	B Precuneus	Shared	—	475	6, −57, 42	10.16
	Cerebellum	Shared	—	284	3, −57, 0	7.88

### Classification

On the basis of the ROC analyses, we determined the classification power of the clusters showing significant group effects in the lFCS and sFCS (see Table 3). We can see that the lFCS and sFCS values in most of these clusters can distinguish the BD patients from the MDD patients, the BD patients from the controls, and the MDD patients from the controls (*p* < 0.05). In particular, the lFCS of the right MTG showed the highest classification power for distinguishing the BD patients from the MDD patients and the BD patients from the controls (*p* < 0.05 and AUC > 0.7). Additionally, the sFCS of the bilateral precuneus showed the highest classification power for distinguishing the MDD patients from the controls (*p* < 0.05 and AUC > 0.7).

**Table 3:** The classification results for the brain clusters showing a significant group effect in the FCS (*p* < 0.001 Alphasim corrected, ANCOVA). The ‘BD’ type indicates that the FCS in the cluster was altered specifically in the BD patients but not in the MDD patients. The ‘shared’ type indicates that the cluster with altered FCS was detected in both the BD and the MDD patients. Abbreviations: lFCS (sFCS), long-range (short-range) functional connectivity strength; BD (MDD), bipolar (major depressive) disorder; MTG, middle temporal gyrus; AUC: area under the ROC curve; B (R), bilateral (right).

FCS	Clusters	Type	BD *vs* MDD		MDD *vs* Controls		BD *vs* Controls	
			AUC	*p*-value	AUC	*p*-value	AUC	*p*-value
lFCS								
	R MTG	BD	0.73	3.2e-6	0.55	2.1e-1	0.79	4.1e-10
	Cerebellum	Shared	0.70	8.2e-5	0.55	1.8e-1	0.63	1.2e-2
sFCS								
	B Thalamus	BD	0.70	7.3e-5	0.54	2.3e-1	0.66	1.8e-3
	B Precuneus	Shared	0.56	1.7e-1	0.69	3.0e-4	0.73	2.91e-6
	Cerebellum	Shared	0.70	7.4e-5	0.62	1.6e-2	0.60	1.6e-2

## Discussion

In a substantial sample of unmedicated adult patients with BD and MDD during a depressive episode, we examined the topological properties of voxel-wise whole-brain functional networks by estimating lFCS and sFCS. Both the BD and MDD patients showed altered lFCS and sFCS in the precuneus and cerebellum. Specific to the BD patients, we found increased lFCS in the MTG and increased sFCS in the bilateral thalamus. The BD group showed more pronounced lFCS and sFCS differences from the controls than the MDD group. These findings provide new evidence for shared and specific neuropathological mechanisms underlying BD and MDD from the perspective of the functional connectivity pattern.

### Shared FCS patterns in BD and MDD

In this study, we found both the BD and MDD patients showed similarly decreased sFCS in the bilateral precuneus (Fig. 2, Table 2), which suggests shared short-range functional connectivity impairments in the precuneus in both disorders. Further ROC analyses indicated that the sFCS of the precuneus could be applied as a candidate biomarker to distinguish MDD patients from controls (Table 3). Nevertheless, a larger sample size is warranted in future studies to replicate our results and to extend the generalizability of our results. These results are consistent with a recent voxel-wise R-fMRI study^36^, which showed decreased short-range functional connectivity in the bilateral precuneus in first-episode, drug-naive adult MDD patients. The precuneus and PCC, the major hub nodes of the DMN, are related to a wide range of cognitive functions, such as episodic memory retrieval, modulation of emotionally salient memories, self-consciousness, and visuo-spatial imagery^37^. Several R-fMRI studies also indicated involvement of the precuneus/PCC in mood disorders, including abnormal spontaneous activity^38^ and functional connectivity^28, 39–42^. Previous task-fMRI studies also reported abnormal activity in the precuneus/PCC in BD and MDD patients during cognitive and emotional tasks^43–45^. Furthermore, other studies found normalized function in the precuneus/PCC in depressive patients following treatment with antidepressant medications^46^ or cognitive behavioral therapy^47^. Taken together, the finding of decreased sFCS in the precuneus suggests that disturbed functional connectivity in the precuneus/PCC could partially contribute to impaired integration of emotion and memory in BD and MDD patients in a depressive episode.

Although the cerebellum is primarily thought to modulate movement, in fact regions of the cerebellum are closely interconnected with the frontal cortices and limbic system^48^, suggesting that it plays a role in the regulation of emotion, affect, and cognitive processes^49, 50^. This study identified increased lFCS and sFCS in the BD patients, but decreased lFCS and sFCS in the MDD patients in the cerebellum (including the anterior, posterior, and vermis) (Figs 1 and 2, Table 2), suggesting altered functional connectivity in the cerebellum in both disorders. These results are in line with two recent studies, which also found reduced FCS in the cerebellum in MDD patients with a history of childhood maltreatment^18^ and increased FCS in the anterior lobe of the cerebellum in BD patients^51^, but these two studies did not classify the FCS into short-range and long-range FCS. Cerebellar abnormalities have been reported in both BD and MDD patients, including abnormalities of gray matter volume^11, 50, 52^, functional activity^28, 38, 50^, functional connectivity^28, 53^, and glucose metabolism^54, 55^. In addition, we observed that the lFCS of the cerebellum was associated with the number of episodes and duration of illness in the BD patients (Fig. 3). Although a longitudinal study will be needed to reach a definitive conclusion, this finding would be compatible with a post-onset, time-dependent progression of the brain changes in BD^56^. Taken together, our finding of an abnormal cerebellum provides additional evidence for the involvement of cerebellar dysfunction in the pathophysiology of BD and MDD.

### Specific FCS patterns in BD

In this study, we found increased lFCS in the right MTG in the BD patients but not in the MDD patients (Fig. 1, Table 2), suggesting BD-specific functional connectivity abnormalities in the MTG. Further ROC analyses indicated that the lFCS of the right MTG may be a candidate biomarker to distinguish BD from MDD and from controls at the highest classification power (Table 3). Increased functional connectivity is typically interpreted as compensatory reallocation or dedifferentiation^57–59^. Several structural MRI studies of BD patients found decreased cortical thickness in temporal regions, including the superior, middle, and inferior temporal gyri^60–62^. Taken together, our results suggest that increased FCS in BD could be a compensatory response to structural deficits. The middle and inferior temporal gyri, located on the lateral surface of the temporal neocortex, are involved in cognitive functions such as language, visual perception, and memory^63–66^. For example, a previous fMRI study found decreased activation in the middle/inferior temporal gyrus in euthymic BD patients during a counting Stroop interference task^66^. Additionally, the middle temporal gyrus (BA 21) has consistently been reported as being part of the DMN^67^. Therefore, our results suggest that abnormal functional connectivity in the MTG might play an important role in the pathophysiological mechanisms of BD and might be associated with neurocognitive deficits.

We also detected increased sFCS in the thalamus in BD in combination with increased lFCS and sFCS in the cerebellum, findings which would suggest disturbed integrity of the cerebello-thalamo-prefrontal functional connectivity in the resting-state in BD. The thalamus, as a central relay station of the brain that filters and gates sensory inputs to the cerebral cortex, is an important key node of the cerebello-thalamo-prefrontal pathway^68^. A recent R-fMRI study found increased functional connectivity between the orbitofrontal cortex and the thalamus in medication-free adolescents with BD^69^. A systematic review of fMRI studies found increased responsiveness in the thalamus in BD but not MDD using facial affect processing paradigms. Structural MRI studies found that BD patients and their unaffected first-degree relatives had smaller thalamus volumes^71, 72^, suggesting that such abnormalities may reflect an underlying vulnerability marker of illness in BD. Evidence from MRI studies showed that structural alteration in the cerebello-thalamo-prefrontal network was associated with neurological soft signs (NSS) in schizophrenia and related psychotic disorders, making this network a candidate for the location of cognitive dysmetria^68, 73^. NSS are minor neurological abnormalities that include motor, sensory, and inhibitory dysfunctions^73^. Previous studies found an increase in NSS in BD patients^73–75^ but not in MDD patients^73^. Taken together, the impairment of the cerebello-thalamo-prefrontal pathway may cause dysfunction of information transmission and functional integration, which may bring about NSS in BD.

### Limitations

This study has several limitations. First, we did not examine data from remitted BD or MDD patients. Therefore, we cannot infer whether the observed group differences persisted once the depressive episodes remitted or whether the findings represent effects of state or trait on the respective illnesses. Second, because we lacked longitudinal data, although the MDD patients in the current study had no family history of BD, we cannot predict whether some patients will later switch to BD. Finally, because clinical symptomatology can change over a period of seven days, collecting the measurements of clinical variables such as the HAMD and YMRS scores, over a seven-day period is another limitation.

In conclusion, this study applied a data-driven, unbiased search for the voxel-wise whole-brain intrinsic functional connectivity in BD and MDD patients as well as in a group of healthy controls. Our results indicated similarly disrupted functional connectivity in the precuneus and cerebellum in BD and MDD. Moreover, we found BD-specific functional connectivity abnormalities in the MTG and thalamus as well as that the BD patients experienced more functional impairment than the MDD patients. Our findings suggest that these two disorders may have some shared and a greater number of specific impairments in the functional connectivity patterns during depressive episodes. These findings provide new evidence for the pathophysiology of BD and MDD at the large-scale whole brain connectivity level.

## Electronic supplementary material

Supplementary Materials

## References

[1] Fountoulakis KN (2010). The emerging modern face of mood disorders: a didactic editorial with a detailed presentation of data and definitions. Ann Gen Psychiatry.

[2] Cardoso de Almeida JR, Phillips ML (2013). Distinguishing between unipolar depression and bipolar depression: current and future clinical and neuroimaging perspectives. Biol Psychiatry.

[3] Bowden CL (2010). Diagnosis, treatment, and recovery maintenance in bipolar depression. J Clin Psychiatry.

[4] Kempton MJ (2011). Structural neuroimaging studies in major depressive disorder. Meta-analysis and comparison with bipolar disorder. Arch Gen Psychiatry.

[5] Fornito A, Bullmore ET (2012). Connectomic intermediate phenotypes for psychiatric disorders. Front Psychiatry.

[6] Serafini G (2014). Brain changes in early-onset bipolar and unipolar depressive disorders: a systematic review in children and adolescents. Eur Child Adolesc Psychiatry.

[7] Versace A (2010). Right orbitofrontal corticolimbic and left corticocortical white matter connectivity differentiate bipolar and unipolar depression. Biol Psychiatry.

[8] Gong Q, He Y (2015). Depression, neuroimaging and connectomics: a selective overview. Biol Psychiatry.

[9] Kaiser RH, Andrews-Hanna JR, Wager TD, Pizzagalli DA (2015). Large-Scale Network Dysfunction in Major Depressive Disorder: A Meta-analysis of Resting-State Functional Connectivity. JAMA Psychiatry.

[10] Vargas C, Lopez-Jaramillo C, Vieta E (2013). A systematic literature review of resting state network–functional MRI in bipolar disorder. J Affect Disord.

[11] Grieve SM, Korgaonkar MS, Koslow SH, Gordon E, Williams LM (2013). Widespread reductions in gray matter volume in depression. Neuroimage Clin.

[12] Zhao YJ (2014). Brain grey matter abnormalities in medication-free patients with major depressive disorder: a meta-analysis. Psychol Med.

[13] Buckner RL (2009). Cortical hubs revealed by intrinsic functional connectivity: mapping, assessment of stability, and relation to Alzheimer’s disease. J Neurosci.

[14] Liang X, Zou Q, He Y, Yang Y (2013). Coupling of functional connectivity and regional cerebral blood flow reveals a physiological basis for network hubs of the human brain. Proc Natl Acad Sci USA.

[15] Tomasi D, Volkow ND (2010). Functional connectivity density mapping. Proc Natl Acad Sci USA.

[16] Vaishnavi SN (2010). Regional aerobic glycolysis in the human brain. Proc Natl Acad Sci USA.

[17] Guo, W. *et al*. Decreased long- and short-range functional connectivity at rest in drug-naive major depressive disorder. *Aust N Z J Psychiatry* 50, 763–769, doi:0004867415617835 (2016). 10.1177/0004867415617835 26607302

[18] Wang L (2014). Overlapping and segregated resting-state functional connectivity in patients with major depressive disorder with and without childhood neglect. Hum Brain Mapp.

[19] Wang L (2015). The effects of antidepressant treatment on resting-state functional brain networks in patients with major depressive disorder. Hum Brain Mapp.

[20] Dai Z (2015). Identifying and Mapping Connectivity Patterns of Brain Network Hubs in Alzheimer’s Disease. Cereb Cortex.

[21] Wang X (2014). Disrupted resting-state functional connectivity in minimally treated chronic schizophrenia. Schizophr Res.

[22] Beucke JC (2013). Abnormally high degree connectivity of the orbitofrontal cortex in obsessive-compulsive disorder. JAMA Psychiatry.

[23] Achard S, Salvador R, Whitcher B, Suckling J, Bullmore E (2006). A resilient, low-frequency, small-world human brain functional network with highly connected association cortical hubs. J Neurosci.

[24] He Y, Chen ZJ, Evans AC (2007). Small-world anatomical networks in the human brain revealed by cortical thickness from MRI. Cereb Cortex.

[25] Guo S (2014). Anatomical distance affects functional connectivity in patients with schizophrenia and their siblings. Schizophr Bull.

[26] Bullmore E, Sporns O (2012). The economy of brain network organization. Nat Rev Neurosci.

[27] Fair DA (2007). Development of distinct control networks through segregation and integration. Proc Natl Acad Sci USA.

[28] Wang Y (2015). Interhemispheric resting state functional connectivity abnormalities in unipolar depression and bipolar depression. Bipolar Disord.

[29] Zuo XN (2012). Network centrality in the human functional connectome. Cereb Cortex.

[30] Fox MD, Zhang D, Snyder AZ, Raichle ME (2009). The global signal and observed anticorrelated resting state brain networks. J Neurophysiol.

[31] Murphy K, Birn RM, Handwerker DA, Jones TB, Bandettini PA (2009). The impact of global signal regression on resting state correlations: are anti-correlated networks introduced?. Neuroimage.

[32] Wang JH (2011). Graph theoretical analysis of functional brain networks: test-retest evaluation on short- and long-term resting-state functional MRI data. PLoS One.

[33] Weissenbacher A (2009). Correlations and anticorrelations in resting-state functional connectivity MRI: a quantitative comparison of preprocessing strategies. Neuroimage.

[34] Ledberg A, Akerman S, Roland PE (1998). Estimation of the probabilities of 3D clusters in functional brain images. Neuroimage.

[35] Yan CG, Wang XD, Zuo XN, Zang YF (2016). DPABI: Data Processing & Analysis for (Resting-State) Brain Imaging. Neuroinformatics.

[36] Ke Z (2016). Abnormal functional connectivity density in first-episode, drug-naive adult patients with major depressive disorder. J Affect Disord.

[37] Cavanna AE, Trimble MR (2006). The precuneus: a review of its functional anatomy and behavioural correlates. Brain.

[38] Liang MJ (2013). Identify changes of brain regional homogeneity in bipolar disorder and unipolar depression using resting-state FMRI. PLoS One.

[39] Greicius, M. D. *et al*. Resting-state functional connectivity in major depression: abnormally increased contributions from subgenual cingulate cortex and thalamus. *Biol Psychiatry* **62**, 429-437, doi:S0006-3223(06)01193-0 (2007). 10.1016/j.biopsych.2006.09.020 PMC2001244 17210143

[40] Hermesdorf M (2016). Major depressive disorder: Findings of reduced homotopic connectivity and investigation of underlying structural mechanisms. Hum Brain Mapp.

[41] Liu Y (2015). Altered effective connectivity model in the default mode network between bipolar and unipolar depression based on resting-state fMRI. J Affect Disord.

[42] Zhu X (2012). Evidence of a dissociation pattern in resting-state default mode network connectivity in first-episode, treatment-naive major depression patients. Biol Psychiatry.

[43] Bradley, K. A. *et al*. Neural correlates of self-perceptions in adolescents with major depressive disorder. *Dev Cogn Neurosci* **19**, 87–97, doi:S1878-9293(15)30053-0 (2016). 10.1016/j.dcn.2016.02.007 PMC4912932 26943454

[44] Malhi GS (2007). Reduced activation to implicit affect induction in euthymic bipolar patients: an fMRI study. J Affect Disord.

[45] Zhang L, Opmeer EM, Ruhe HG, Aleman A, van der Meer L (2015). Brain activation during self- and other-reflection in bipolar disorder with a history of psychosis: Comparison to schizophrenia. Neuroimage Clin.

[46] Wang L (2014). Short-term effects of escitalopram on regional brain function in first-episode drug-naive patients with major depressive disorder assessed by resting-state functional magnetic resonance imaging. Psychol Med.

[47] Franklin G, Carson AJ, Welch KA (2016). Cognitive behavioural therapy for depression: systematic review of imaging studies. Acta Neuropsychiatr.

[48] Ramnani N (2006). The primate cortico-cerebellar system: anatomy and function. Nat Rev Neurosci.

[49] Schmahmann JD (2004). Disorders of the cerebellum: ataxia, dysmetria of thought, and the cerebellar cognitive affective syndrome. J Neuropsychiatry Clin Neurosci.

[50] Phillips JR, Hewedi DH, Eissa AM, Moustafa AA (2015). The cerebellum and psychiatric disorders. Front Public Health.

[51] Wang Y (2016). Disrupted Resting-State Functional Connectivity in Nonmedicated Bipolar Disorder. Radiology.

[52] Redlich R (2014). Brain morphometric biomarkers distinguishing unipolar and bipolar depression. A voxel-based morphometry-pattern classification approach. JAMA Psychiatry.

[53] Guo W (2015). Increased cerebellar-default-mode-network connectivity in drug-naive major depressive disorder at rest. Medicine (Baltimore).

[54] Su L (2014). Cerebral metabolism in major depressive disorder: a voxel-based meta-analysis of positron emission tomography studies. BMC Psychiatry.

[55] Altamura AC (2013). White matter metabolism differentiates schizophrenia and bipolar disorder: a preliminary PET study. Psychiatry Res.

[56] Haukvik UK (2016). No progressive brain changes during a 1-year follow-up of patients with first-episode psychosis. Psychol Med.

[57] Guo W (2015). Increased short-range and long-range functional connectivity in first-episode, medication-naive schizophrenia at rest. Schizophr Res.

[58] Grady, C. L., McIntosh, A. R. & Craik, F. I. Task-related activity in prefrontal cortex and its relation to recognition memory performance in young and old adults. *Neuropsychologia* **43**, 1466–1481, doi:S0028-3932(05)00017-5 (2005). 10.1016/j.neuropsychologia.2004.12.016 15989937

[59] Cabeza, R., Anderson, N. D., Locantore, J. K. & McIntosh, A. R. Aging gracefully: compensatory brain activity in high-performing older adults. *Neuroimage* **17**, 1394–1402, doi:S1053811902912802 (2002). 10.1006/nimg.2002.1280 12414279

[60] Fung G (2015). Distinguishing bipolar and major depressive disorders by brain structural morphometry: a pilot study. BMC Psychiatry.

[61] Hanford LC, Nazarov A, Hall GB, Sassi RB (2016). Cortical thickness in bipolar disorder: a systematic review. Bipolar Disord.

[62] Hozer F, Houenou J (2016). Can neuroimaging disentangle bipolar disorder?. J Affect Disord.

[63] Noppeney U, Price CJ (2002). Retrieval of visual, auditory, and abstract semantics. Neuroimage.

[64] Ojemann GA, Schoenfield-McNeill J, Corina DP (2002). Anatomic subdivisions in human temporal cortical neuronal activity related to recent verbal memory. Nat Neurosci.

[65] Price CJ (2000). The anatomy of language: contributions from functional neuroimaging. J Anat.

[66] Strakowski SM (2005). Abnormal FMRI brain activation in euthymic bipolar disorder patients during a counting Stroop interference task. Am J Psychiatry.

[67] Pievani M (2017). Coordinate-Based Meta-Analysis of the Default Mode and Salience Network for Target Identification in Non-Invasive Brain Stimulation of Alzheimer’s Disease and Behavior Variant Frontotemporal Dementia Networks. J Alzheimers Dis.

[68] Mouchet-Mages S (2011). Correlations of cerebello-thalamo-prefrontal structure and neurological soft signs in patients with first-episode psychosis. Acta Psychiatr Scand.

[69] Son, Y. D., Han, D. H., Kim, S. M., Min, K. J. & Renshaw, P. F. A functional connectivity comparison between attention deficit hyperactivity disorder and bipolar disorder in medication-naive adolescents with mood fluctuation and attention problems. *Psychiatry Res* **263**, 1–7, doi:S0925-4927(16)30233-5 (2017). 10.1016/j.pscychresns.2017.02.006 28264765

[70] Delvecchio G (2012). Common and distinct neural correlates of emotional processing in Bipolar Disorder and Major Depressive Disorder: a voxel-based meta-analysis of functional magnetic resonance imaging studies. Eur Neuropsychopharmacol.

[71] Hibar DP (2016). Subcortical volumetric abnormalities in bipolar disorder. Mol Psychiatry.

[72] Nery FG, Monkul ES, Lafer B (2013). Gray matter abnormalities as brain structural vulnerability factors for bipolar disorder: A review of neuroimaging studies of individuals at high genetic risk for bipolar disorder. Aust N Z J Psychiatry.

[73] Zhao Q (2014). Neurological soft signs are not “soft” in brain structure and functional networks: evidence from ALE meta-analysis. Schizophr Bull.

[74] Goswami U (2006). Neuropsychological dysfunction, soft neurological signs and social disability in euthymic patients with bipolar disorder. Br J Psychiatry.

[75] Mrad A, Wassim Krir M, Ajmi I, Gaha L, Mechri A (2016). Neurological soft signs in euthymic bipolar I patients: A comparative study with healthy siblings and controls. Psychiatry Res.

