# Supplementary material for: Shared and Specific Intrinsic Functional Connectivity Patterns in Unmedicated Bipolar Disorder and Major Depressive Disorder
Source: Sci Rep. 2017 Jun 15;7:3570. doi: 10.1038/s41598-017-03777-8 (PMC5472613; doi:10.1038/s41598-017-03777-8)
Supplement: Supplementary file 1 — Supplementary Materials [file 41598_2017_3777_MOESM1_ESM.doc]

**Supplementary Materials**

**Shared and Specific Intrinsic Functional Connectivity Patterns in Unmedicated Bipolar Disorder and Major Depressive Disorder**

Ying Wang^1,2^*^†^, Junjing Wang^3^*, Yanbin Jia^4^, Shuming Zhong^4^, Meiqi Niu^3^, Yao Sun^1^, Zhangzhang Qi^1^, Ling Zhao^3^, Li Huang^1^, Ruiwang Huang^3†^

^1^ Medical Imaging Center, First Affiliated Hospital of Jinan University, Guangzhou 510630, China

^2^ Clinical Experimental Center, First Affiliated Hospital of Jinan University, Guangzhou 510630, China

^3^ Center for the Study of Applied Psychology & MRI Center, Key Laboratory of Mental Health and Cognitive Science of Guangdong Province, School of Psychology, Institute of Brain Research and Rehabilitation, South China Normal University, Guangzhou 510631, China

^4^ Department of Psychiatry, First Affiliated Hospital of Jinan University, Guangzhou 510630, China

* These authors contributed equally to this work.

^†^ Correspondence should be addressed to:

Ruiwang Huang, Ph.D.

School of Psychology

South China Normal University, Guangzhou, 510631, China

Tel/Fax: (+86)20 – 8521 6499

E-mail: [ruiwang.huang@gmail.com](mailto:ruiwang.huang@gmail.com)

Ying Wang, Ph.D.

Medical Imaging Center, First Affiliated Hospital of Jinan University,

Guangzhou 510630, China.

Tel.: (+86) 20 – 38688071

E-mail: [johneil@vip.sina.com](mailto:johneil@vip.sina.com)

**Supplementary Materials**

**Shared and Specific Intrinsic Functional Connectivity Patterns in Unmedicated Bipolar Disorder and Major Depressive Disorder**

***Participant Medication Status***

Fifteen of the BD II and 24 of the MDD patients were medication naïve; the others had not received medication for at least five months before the time of scanning and were therefore currently unmedicated. The patients who were medication naïve were so because they had never been diagnosed or did not want to take medication. In addition, the included patients had generally visited their physician (psychiatrist/general practitioner) because of a depressive relapse after quitting medication. Thirty-three patients with BD had previously been treated with antidepressants (duloxetine or paroxetine), and/or mood stabilizers (lithium, sodium valproate), and/or atypical antipsychotic medications (olanzapine or risperidone), and 24 patients with MDD had previously been treated with antidepressants (duloxetine or paroxetine) but had been off medication for at least 5 months prior to the scan. Importantly, all the enrolled participants were already medication-free at the time of their initial screening to determine eligibility and, thus, were never encouraged to cease medication to take part in the study. After we finished conducting the MRI and the HAMD measurement, all the out- or in-patients with BD and MDD were treated with psychotropic medication.

**Robustness**

To evaluate the robustness of our results, we repeated the network analyses using the following procedures.

*The effects of different correlation thresholds*

When computing the FCS, we used a correlation coefficient threshold of *r_ij_* > 0.3307 (*p* < 0.05, Bonferroni correction) to eliminate weak correlations possibly arising from signal noise. To test whether our main results depended on the selection of the correlation thresholds, we repeated the network analyses by selecting two other widely used correlation thresholds of *r_ij_* > 0.25 and *r_ij_* > 0.4 .

*The effects of different preprocessing choices*

Several studies suggested that the global signal may be associated with respiration and should be removed in R-fMRI analysis to reduce the effects of physiological artifacts [^5^](#_ENREF_5). Moreover, several other studies proposed that removing the global signal may introduce widespread negative functional connectivity and thus alter the intrinsic correlation structure of brain networks. In addition, a recent fMRI study suggested that the global signal may be associated with an oscillatory neuronal signal and may have biological meaning [^7^](#_ENREF_7). To test the robustness of our results, we re-analyzed our data without regressing out the global signal.

*The effects of network members*

Considering the disagreements in treating negative correlations in the R-fMRI studies, we also repeated the FCS analyses by taking negative and absolute correlations, separately [^8^](#_ENREF_8).

*The effects of network types*

Although analyzing the weighted FCS can accurately characterize the brain architecture, this type of FCS may be sensitive to noise and therefore be less reliable [^9^](#_ENREF_9). Thus, we repeated the FCS analyses in the current study using the binary functional network [^8^](#_ENREF_8).

**Results**

The validation analyses, in which we selected different analytical strategies, including different correlation thresholds (Tables S1 and S2 in Supplementary materials), preprocessing choices (Table S3 in Supplementary materials), network members (Table S4 and S5 in Supplementary materials), and network types (Table S6 in Supplementary materials), indicated that our main results were reasonably stable. All showed that the BD and MDD groups had shared and specific impairments in their FCS values during a depressive period. Moreover, the BD group showed more pronounced FCS alterations than the MDD group.

**Statistical analyses**

To compare with previous studies on alterations in depression disorders, we also conducted two sample t-tests for the lFCS and sFCS between the BD patients and the controls, the MDD patients and the controls, as well as between the BD and the MDD patients. The results can be found in Figs. S3 and S4.

**Supplementary Figures**

**Figure S1** The group average long-range functional connectivity strength (lFCS). (a) For the patients with bipolar disorder (BD), (b) for the patients with major depressive disorder (MDD), and (c) for the healthy controls. The color bar indicates the lFCS values.


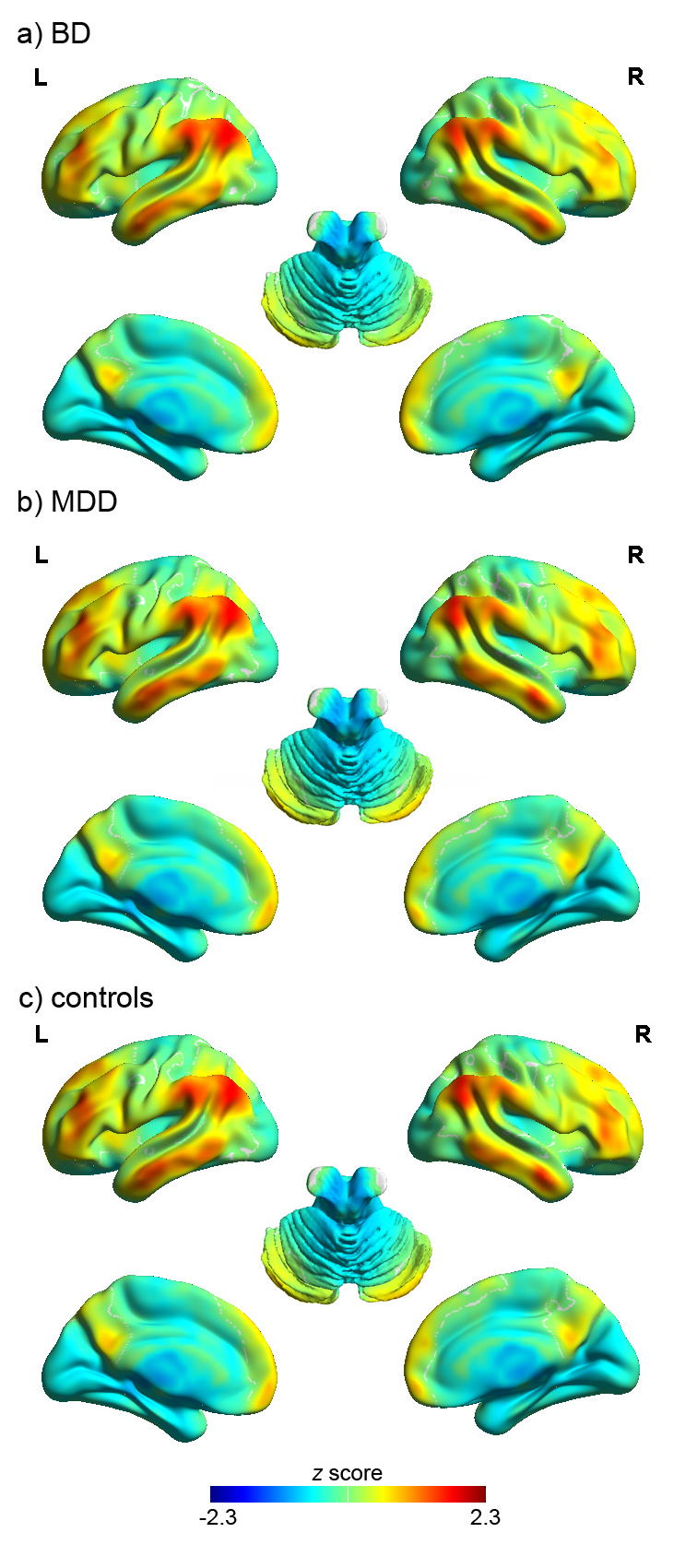


**Figure S2** The group average short-range functional connectivity strength (sFCS). (a) For the patients with bipolar disorder (BD), (b) for the patients with major depressive disorder (MDD), and (c) for the healthy controls. The color bar indicates the sFCS values.

**
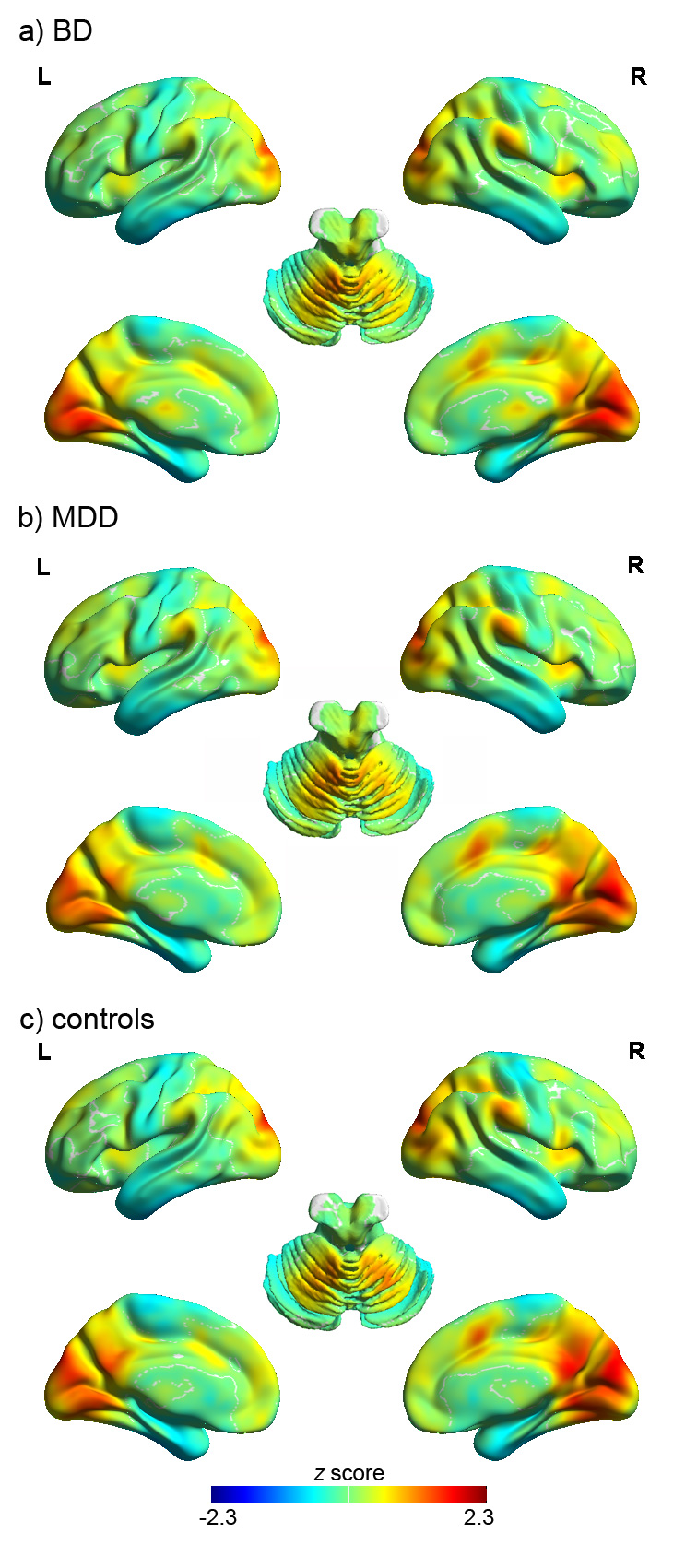
**

**Figure S3** Statistical results of the lFCS (*p* < 0.001 Alphasim corrected, two sample *t*-test). (a) BD patients versus controls, and (b) BD patients versus MDD patients. There were no significant differences between the MDD patients and the controls. Abbreviations: lFCS: long-range functional connectivity strength; BD (MDD), bipolar (major depressive) disorder; MTG, middle temporal gyrus; STG, superior temporal gyrus; B (L, R), bilateral (left, right).


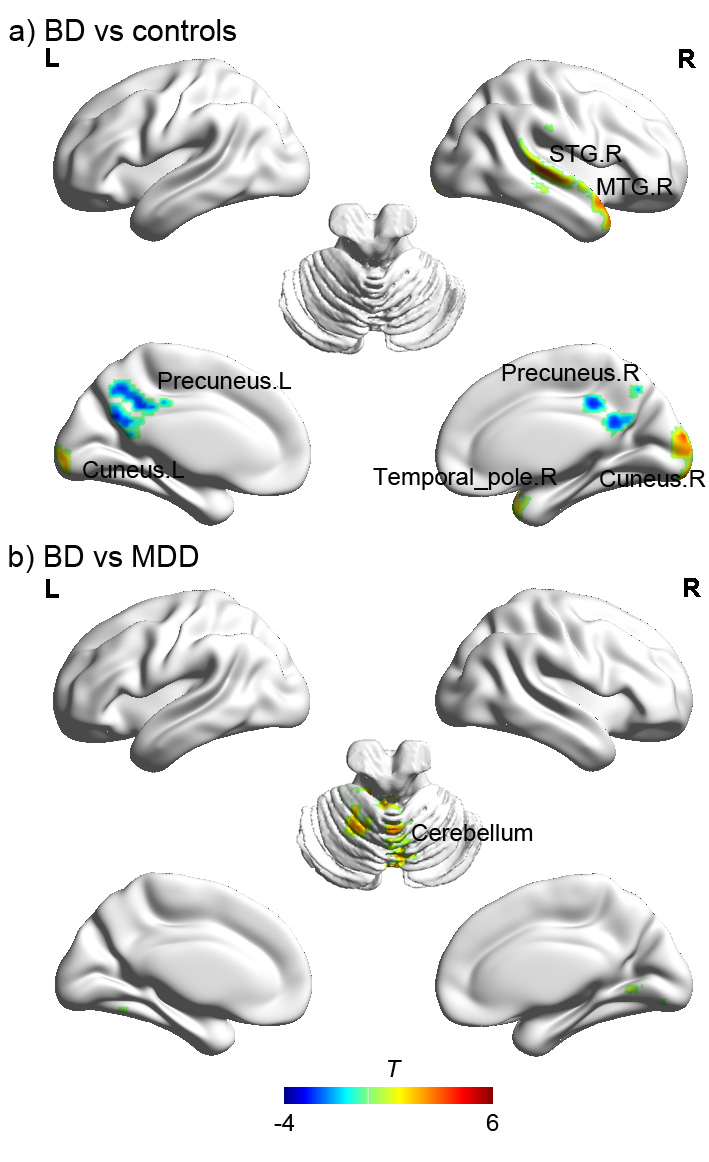


**Figure S4** Statistical results of the sFCS (*p* < 0.001 Alphasim corrected, two sample *t*-test). (a) BD patients versus controls, (b) MDD patients versus controls, and (c) BD patients versus MDD patients. Abbreviations: sFCS: short-range functional connectivity strength; BD (MDD), bipolar (major depressive) disorder; SFG, superior frontal gyrus; MFG, middle frontal gyrus B (L, R), bilateral (left, right).


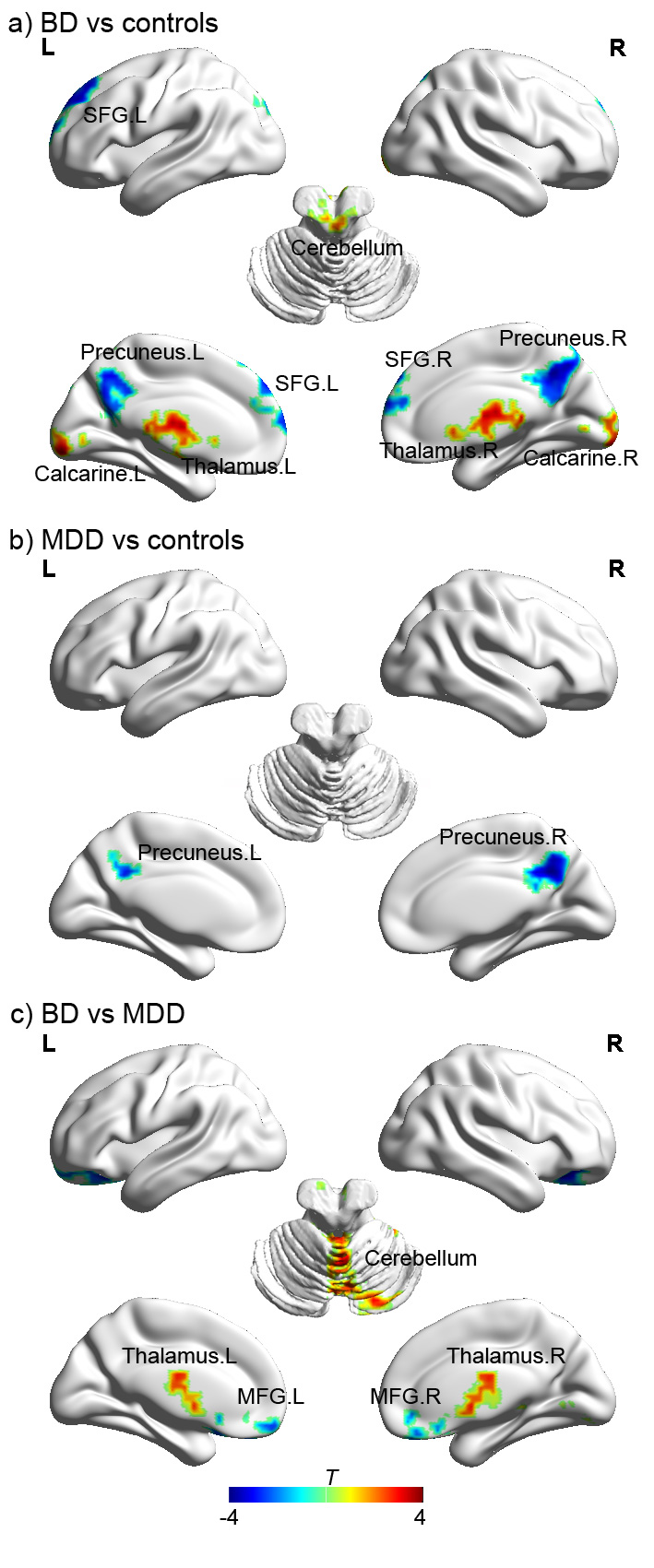


**Supplementary Tables**

**Table S1** Brain clusters showing a significant group effect in the FCS when using *r*_ij_ > 0.25 to threshold the inter-voxel correlations (*p* < 0.001 Alphasim corrected, ANCOVA). The ‘BD’ type indicates that the FCS in the cluster was specifically altered in the BD patients but not in the MDD patients. The ‘shared’ type indicates that the cluster with altered FCS was detected in both the BD and the MDD patients. Abbreviations: lFCS (sFCS), long-range (short-range) functional connectivity strength; BD (MDD), bipolar (major depressive) disorder; MTG, middle temporal gyrus; STG, superior temporal gyrus; B (L, R), bilateral (left, right).

| **FCS** | **Cluster** | **Type** | **Location** | **Cluster size (voxels)** | **MNI coordinates**  **(x, y, z)** | ***F-*value** |
| --- | --- | --- | --- | --- | --- | --- |
| lFCS |  |  |  |  |  |  |
|  | R MTG | BD | BA 21 | 356 | 66, -27, -3 | 18.06 |
|  | Cerebellum | Shared | - | 548 | 21, -54, -45 | 7.95 |
|  |  |  |  |  |  |  |
| sFCS |  |  |  |  |  |  |
|  | B Thalamus | BD | - | 313 | 9, -12, 12 | 9.06 |
|  | L SFG | BD | BA 9 | 335 | -18, 45, 30 | 8.29 |
|  |  |  |  |  |  |  |
|  | Cerebellum vermis | Shared | BA 18 | 365 | 3, -57, -3 | 7.25 |
|  | B Precuneus | Shared | - | 377 | 6, -57, 42 | 10.29 |

**Table S2** Brain clusters showing a significant group effect in the FCS when using *r*_ij_ > 0.4 to threshold the inter-voxel correlations (*p* < 0.001 Alphasim corrected, ANCOVA). The ‘BD’ type indicates that the FCS in the cluster was altered specifically in the BD patients but not in the MDD patients. The ‘shared’ type indicates that the cluster with an altered FCS was detected in both the BD and the MDD patients. Abbreviations: lFCS (sFCS), long-range (short-range) functional connectivity strength; BD (MDD), bipolar (major depressive) disorder; STG, superior temporal gyrus; B (R), bilateral (right).

| **FCS** | **Cluster** | **Type** | **Location** | **Cluster size (voxels)** | | **MNI coordinates**  **(x, y, z)** | ***F-*value** |
| --- | --- | --- | --- | --- | --- | --- | --- |
| lFCS |  |  |  | |  |  |  |
|  | R STG | BD | BA 22 | | 313 | -63, -12, -3 | 12.06 |
|  | Cerebellum | Shared | - | | 303 | 9, -60, -33 | 7.81 |
|  |  |  |  | |  |  |  |
| sFCS |  |  |  | |  |  |  |
|  | B Precuneus | Shared | - | | 543 | 6, -57, 42 | 9.24 |

**Table S3** Brain clusters showing significant group effects in the FCS when preprocessing the R-fMRI data without regressing out the global signal (*p* < 0.001 Alphasim corrected, ANCOVA). The ‘shared’ type indicates that the cluster with an altered FCS was detected in both the BD and the MDD patients. Abbreviations: lFCS (sFCS), long-range (short-range) functional connectivity strength; BD (MDD), bipolar (major depressive) disorder; IPG, inferior parietal gyrus; B (L), bilateral (left).

| **FCS** | **Cluster** | **Type** | **Location** | **Cluster size (voxels)** | **MNI coordinates**  **(x, y, z)** | ***F-*value** | |
| --- | --- | --- | --- | --- | --- | --- | --- |
| lFCS |  |  |  |  |  |  | |
|  | L IPG | Shared | BA 7 | 277 | -21, -69, 39 | 9.35 | |
|  | | | | | | |  |

**Table S4** Brain clusters showing significant group effects in the FCS when adopting absolute inter-voxel correlations (*p* < 0.001 Alphasim corrected, ANCOVA). The ‘BD’ type indicates that the FCS in the cluster was altered specifically in the BD patients but not in the MDD patients. The ‘MDD’ type indicates that the FCS in the cluster was altered specifically in the MDD patients but not in the BD patients. The ‘shared’ type indicates that the cluster with altered FCS was detected in both the BD and the MDD patients. Abbreviations: lFCS (sFCS), long-range (short-range) functional connectivity strength; BD (MDD), bipolar (major depressive) disorder; MTG, middle temporal gyrus; B (L, R), bilateral (left, right).

| **FCS** | **Cluster** | **Type** | **Location** | **Cluster size (voxels)** | **MNI coordinates**  **(x, y, z)** | ***F-*value** |
| --- | --- | --- | --- | --- | --- | --- |
| lFCS |  |  |  |  |  |  |
|  | R MTG | BD | BA 21 | 293 | 66, -27, -3 | 15.19 |
|  |  |  |  |  |  |  |
|  | L Calcarine | MDD | BA 17 | 632 | -3, -99, 0 | 10.11 |
|  |  |  |  |  |  |  |
| sFCS |  |  |  |  |  |  |
|  | B Thalamus | BD | - | 299 | 9, -12, 12 | 8.91 |
|  |  |  |  |  |  |  |
|  | B Precuneus | Shared | - | 585 | 6, -57,42 | 9.53 |
|  | Cerebellum | Shared | - | 452 | 0, -54, -15 | 8.63 |

**Table S5** Brain clusters showing significant group effects in the FCS when including the negative inter-voxel correlations (*p* < 0.001 Alphasim corrected, ANCOVA). The ‘BD’ type indicates that the FCS in the cluster was altered specifically in the BD patients but not in the MDD patients. The ‘MDD’ type indicates that the FCS in the cluster was altered specifically in the MDD patients but not in the BD patients. The ‘shared’ type indicates that the cluster with an altered FCS was detected in both the BD and the MDD patients. Abbreviations: lFCS (sFCS), long-range (short-range) functional connectivity strength; BD (MDD), bipolar (major depressive) disorder; B, bilateral.

| **FCS** | **Cluster** | **Type** | **Location** | **Cluster size (voxels)** | **MNI coordinates**  **(x, y, z)** | ***F-*value** |
| --- | --- | --- | --- | --- | --- | --- |
| lFCS |  |  |  |  |  |  |
|  | B Calcarine | MDD | BA 17 | 293 | -3, -99, 0 | 9.29 |
|  |  |  |  |  |  |  |
| sFCS |  |  |  |  |  |  |
|  | Cerebellum | Shared | / | 819 | 0, -54, -15 | 9.90 |
|  | B Precuneus | Shared | BA 7 | 392 | -18, -75, 51 | 7.56 |

**Table S6** Brain clusters showing significant group effects in the FCS for the binarized functional network (*p* < 0.001 Alphasim corrected, ANCOVA). The ‘BD’ type indicates that the FCS in the cluster was altered specifically in the BD patients but not in the MDD patients. The ‘MDD’ type indicates that the FCS in the cluster was altered specifically in the MDD patients but not in the BD patients. The ‘shared’ type indicates that the cluster with altered FCS was detected in both the BD and the MDD patients. Abbreviations: lFCS (sFCS), long-range (short-range) functional connectivity strength; BD (MDD), bipolar (major depressive) disorder; MTG, middle temporal gyrus; SFG, superior frontal gyrus; B (R), bilateral (right).

| **FCS** | **Cluster** | **Type** | **Location** | **Cluster size (voxels)** | **MNI coordinates**  **(x, y, z)** | ***F-*value** |
| --- | --- | --- | --- | --- | --- | --- |
| lFCS |  |  |  |  |  |  |
|  | R MTG | BD | BA 21 | 325 | 66, -27, -3 | 16.21 |
|  |  |  |  |  |  |  |
|  | Cerebellum | Shared | / | 531 | -6, -48, -24 | 7.92 |
|  |  |  |  |  |  |  |
| sFCS |  |  |  |  |  |  |
|  | B thalamus | BD | / | 304 | 9, -12, 12 | 9.05 |
|  | B SFG | BD | BA 32 | 329 | -18, 42, 37 | 329 |
|  |  |  |  |  |  |  |
|  | Cerebellum | Shared | / | 386 | 15, -81, -18 | 7.57 |
|  | B Precuneus | Shared | / | 417 | 6, -57, 42 | 10.00 |

**References**

1 Li, S. *et al.* Abnormal degree centrality in neurologically asymptomatic patients with end-stage renal disease: A resting-state fMRI study. *Clin Neurophysiol*(2015).

2 Tomasi, D. & Volkow, N. D. Mapping small-world properties through development in the human brain: disruption in schizophrenia. *PLoS One* **9**, e96176(2014).

3 Birn, R. M., Diamond, J. B., Smith, M. A. & Bandettini, P. A. Separating respiratory-variation-related fluctuations from neuronal-activity-related fluctuations in fMRI. *Neuroimage* **31**, 1536-1548(2006).

4 Chang, C. & Glover, G. H. Effects of model-based physiological noise correction on default mode network anti-correlations and correlations. *Neuroimage* **47**, 1448-1459(2009).

5 Fox, M. D., Zhang, D., Snyder, A. Z. & Raichle, M. E. The global signal and observed anticorrelated resting state brain networks. *J Neurophysiol* **101**, 3270-3283(2009).

6 Murphy, K., Birn, R. M., Handwerker, D. A., Jones, T. B. & Bandettini, P. A. The impact of global signal regression on resting state correlations: are anti-correlated networks introduced? *Neuroimage* **44**, 893-905(2009).

7 Scholvinck, M. L., Maier, A., Ye, F. Q., Duyn, J. H. & Leopold, D. A. Neural basis of global resting-state fMRI activity. *Proc Natl Acad Sci U S A* **107**, 10238-10243 (2010).

8 Shen, Y. *et al.* Sub-hubs of baseline functional brain networks are related to early improvement following two-week pharmacological therapy for major depressive disorder. *Hum Brain Mapp* **36**, 2915-2927 (2015).

9 Wang, J. H. *et al.* Graph theoretical analysis of functional brain networks: test-retest evaluation on short- and long-term resting-state functional MRI data. *PLoS One* **6**, e21976, (2011).
